# Concomitant Administration of Different Doses of Simvastatin with Ivabradine Influence on PAI-1 and Heart Rate in Normo- and Hypercholesterolaemic Rats

**DOI:** 10.1100/2012/976519

**Published:** 2012-05-03

**Authors:** Jacek Owczarek, Magdalena Jasińska-Stroschein, Daria Orszulak-Michalak

**Affiliations:** Department of Biopharmacy, Medical University of Lodz, Muszynskiego 1, 90-151 Lodz, Poland

## Abstract

Ivabradine is a novel heart rate lowering agent that inhibits I_f_ ionic current in the sinus node and demonstrates antiischaemic and antianginal activity. The aim of the paper was to investigate the effect its dose-dependent drug-drug interaction with simvastatin inhibitor HMGCo-A has on PAI-1 blood level, heart rate and blood pressure. The experiments were performed in hyper- and normocholesterolemic Wistar rats receiving simvastatin (1 and 20 mg × kg^−1^ bw) with ivabradine (10 mg × kg^−1^ bw) during a 4-week period. Ivabradine exacerbated the decrease of PAI-1 in normocholesterolemic animals receiving simvastatin at a dose of 1 mg/kg bw and was not observed to have any significant influence on the PAI-1 values in rats receiving 20 mg × kg^−1^ bw simvastatin. Ivabradine, coadministered with simvastatin given at a dose of 20 mg × kg^−1^ bw, significantly slowed the heart rate in normocholesterolaemic and hypercholesterolaemic groups as compared to the group receiving ivabradine alone. *Conclusion.* The administration of ivabradine to normocholesterolaemic and hypercholesterolaemic rats receiving simvastatin significantly exacerbated the slowing of heart rate with no effect on blood pressure. The administration of ivabradine has been shown to demonstrate different effects on PAI-1 values depending on lipid disorders.

## 1. Introduction

The resting heart rate value acts as an independent factor of the risk associated with cardiovascular problems [1–3]. A significant advantage of the slowing of the heart rate is connected with reduced demand of the heart muscles for oxygen, as well as a beneficial influence on the function of the blood vessel endothelium [4, 5]. The novel selective for the I_f_ current lowering heart rate agent, specifically slow cardiac frequency, by decreasing the rate of diastolic depolarization [6]. Ivabradine seems to have an additional effect in patients with stabile coronary artery disease (CAD) without and with left ventricular systolic dysfunction (LVSD) [7–9]. Preclinical studies show that inhibition of the HCN channel slows the rhythm to varying degrees in the atria, ventricle, and outflow tract [10]. Ivabradine reduces heart rate in the sinoatrial node without affecting blood pressure or myocardial contractility, intracardiac conduction, or ventricular repolarization [11]. In ischaemic heart disease (IHD) patients, the role played by HMGCo-A inhibitors in the prevention of cardiovascular events is well established. Their beneficial activity is dependent on the limitation of cholesterol synthesis as well as cholesterol-independent “pleiotropic” effects [12]. It has been shown in earlier clinical studies that simvastatin at a dose of 40 mg/day given for a period of 8 weeks significantly reduced the levels of inflammatory markers [13] as well as inhibited the activity of the circulating fibrinolysis inhibitor factor-plasminogen activator inhibitor 1(PAI-1) [14]. Similar observations have been noted in laboratory studies [15]. The influence on fibrinolysis processes were then observed depending on the “mechanism that involves geranylgeranyl-modified intermediates.”

The aim of this paper was to assess the impact of the administration of ivabradine alone and combined with various doses of simvastatin on PAI-1 and heart rate values in normocholesterolaemic and hypercholesterolaemic rats.

## 2. Materials and Methods

### 2.1. Study Protocol

The study was approved by the Ethics Committee of the Medical University of Lodz (Poland)–2/ŁB441/2009. The experiments were performed in 101, Wistar rats, outbred males, 200–240 g bw. An adaptation period lasting several days was scheduled prior to the beginning of the experiment. After the adaptation period, animals were divided into 2 groups: those receiving a normal diet (normocholesterolaemic rats) or those receiving a diet with 5% cholesterol and 2.5% cholic acid (hypercholesterolaemic rats). After a four-week period, each group was divided into 6 subgroups which, for 4 weeks, received intragastric (i.g.) doses of:

1. 0.1% methylcellulose (control group);
2. ivabradine 10 mg × kg^−1^ bw;
3. simvastatin 1 mg × kg^−1^ bw;
4. simvastatin 20 mg × kg^−1^ bw;
5. simvastatin 1 mg × kg^−1^ bw + Ivabradine 10 mg × kg^−1^ bw;
6. simvastatin 20 mg × kg^−1^ bw + Ivabradine 10 mg × kg^−1^ bw.

All rats had free access to food and water throughout the study. After an eight-week period of diet and drug administration, heart rate, and hemodynamic parameters were measured. The surgery was performed 24 h after administration of the last drug dose and 10 h after the last feed supply. For the further surgical procedures, anesthesia was initiated by an intraperitoneal (i.p.) dose of pentobarbital sodium at 60 mg × kg^−1^ bw. The anesthesia was maintained by intraperitoneal bolus injections of pentobarbital sodium at 10 mg × kg^−1^ bw as needed. For the measurements of heart rate, and blood pressure, catheters were implanted into the right carotid artery. The signals were provided by an Isotec pressure transducer connected to a direct current bridge amplifier (both Hugo Sachs Elektronik) for 20 minutes after the hemodynamic parameter stabilization period. For the further PAI-1 assessment and lipid profile examination, blood samples were taken. Surgical procedures, heart rate and blood pressure recording were provided as described previously [16, 17]. Plasma PAI-1 levels were determined using ELISA kits from American Diagnostica following the manufacturer's instructions.

### 2.2. Statistics

All data were presented as means ± SD (standard deviation). Statistical comparisons between the groups were performed using ANOVA, and *post hoc* comparisons were performed using the LSD test. The normal distribution of parameters was checked by means of the Shapiro-Wilks test. If the data was not normally distributed or the values of variance were different, ANOVA with Kruscal-Wallis and Mann-Whitney's *U* test were used. All parameters were considered significantly different if *P* < 0.05. The statistical analysis of heart rate and hemodynamic parameters was performed using Statgraphics 5.0 plus software.

## 3. Results

### 3.1. Lipid Profile

The lipid profiles achieved in normocholesterolaemic and hypercholesterolaemic rats are presented in Tables 1 and 2.

### 3.2. Blood Pressure

Ivabradine and simvastatin given alone or in combination was found to have an insignificant influence on the mean, systolic, and diastolic blood pressure in normocholesterolaemic and hypercholesterolaemic rats (Tables 3 and 4).

### 3.3. Heart Rate

In normocholesterolaemic and hypercholesterolaemic rats receiving simvastatin at doses of 1 and 20 mg × kg^−1^ bw alone, no significant differences were seen in the heart rate disturbances compared to control groups. Ivabradine administration to normocholesterolaemic rats resulted in significant deceleration of heart rate compared to the control group (350.2 ± 16.2 versus 434.8 ± 17.2 min^−1^). Similar results were also observed in the hypercholesterolaemic group (363 ± 21.7 versus 435.3 ± 20.3 min^−1^). The heart rate values after concomitant administration of ivabradine and simvastatin at a dose of 1 mg × kg^−1^ bw to normocholesterolaemic rats were significantly decreased compared to the control group (342.3 ± 28.6 versus 434.8 ± 17.2 min^−1^) and compared to the group receiving simvastatin alone. Similar observations were made in hypercholesterolaemic rats. There were no statistical differences in heart rate concerning concomitant administration of ivabradine and simvastatin at dose 1 mg × kg^−1^ bw between hyper- and normocholesterolaemic rats. Administration of ivabradine with simvastatin at a dose of 20 mg × kg^−1^ bw to hypercholesterolaemic rats significantly reduced heart rate compared to the control group (319.6 ± 30.6 versus 435.3 ± 20.3 min^−1^) and compared to the groups receiving simvastatin at a dose of 1 or 20 mg × kg^−1^ bw alone.

In the normocholesterolaemic group, the slowing of the heart rate was statistically similar to hypercholesterolaemic rats. Administration of ivabradine with simvastatin at a dose of 20 mg × kg^−1^ bw to hypercholesterolaemic and normocholesterolaemic rats similarly decreased heart rate. The concomitant administration of ivabradine with simvastatin at a dose of 20 mg × kg^−1^ bw to hypercholesterolaemic and normocholesterolaemic was shown to significantly decrease the heart rate compared to rats receiving ivabradine alone (Figures 3 and 4).

### 3.4. PAI-1 Blood Level

**Figure 1:**
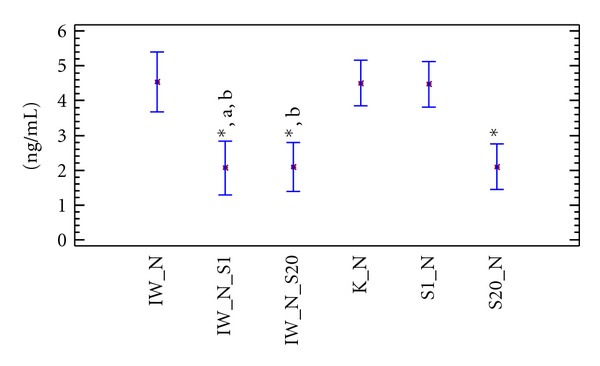
PAI–1 blood level (ng/mL) in Wistar rats fed normocholesterolaemic diet. K_N: normocholesterolaemic control group, IW_N: normocholesterolaemic group receiving ivabradine, S1_N: normocholesterolaemic group receiving simvastatin at a dose of 1 mg × kg^−1^ bw, S20_N: normocholesterolaemic group receiving simvastatin at a dose of 20 mg × kg^−1^ bw, IW_N_S1: normocholesterolaemic group receiving ivabradine and simvastatin at a dose of 1 mg × kg^−1^ bw, IW_N_S20: normocholesterolaemic group receiving ivabradine and simvastatin at a dose of 20 mg × kg^−1^ bw, *P < 0.05 as compared to the control group, (a) *P* < 0.05 as compared to rats receiving simvastatin alone, and (b) *P* < 0.05 as compared to rats receiving ivabradine alone.

In normocholesterolaemic rats, the administration of ivabradine was seen to have no statistically significant influence on PAI-1 values compared to the control group or the group receiving 1 mg/kg bw of simvastatin alone. The administration of simvastatin at dose of 20 mg/kg significantly (*P* < 0.05) reduced the levels of PAI-1 compared to the control group. After a combined dose of ivabradine with 1 or 20 mg × kg^−1^ bw simvastatin, a significant reduction of the level of PAI-1 was seen compared to the group which only received ivabradine (*P* < 0.05) (Figure 1).

**Figure 2:**
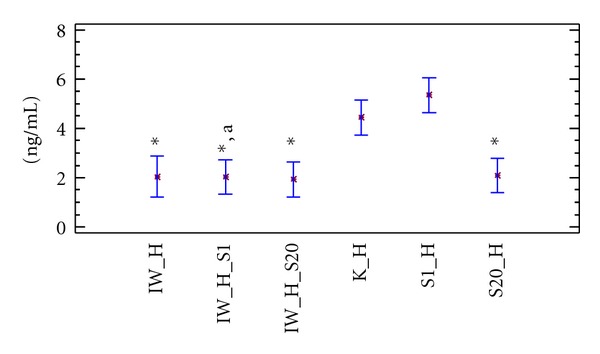
PAI–1 level (ng/mL) in Wistar rats fed hypercholesterolaemic diet. K_H: hypercholesterolaemic control group, IW_H: hypercholesterolaemic group receiving ivabradine, S1_H: hypercholesterolaemic group receiving simvastatin at a dose of 1 mg × kg^−1^ bw, S20_H: hypercholesterolaemic group receiving simvastatin at a dose of 20 mg × kg^−1^ bw, IW_H_S1: hypercholesterolaemic group receiving ivabradine and simvastatin at a dose of 1 mg × kg^−1^ bw, IW_H_S20: hypercholesterolaemic group receiving ivabradine and simvastatin at a dose of 20 mg × kg^−1^ bw, *P < 0.05 as compared to the control group, and (a) *P* < 0.05 as compared to rats receiving simvastatin alone.

**Figure 3:**
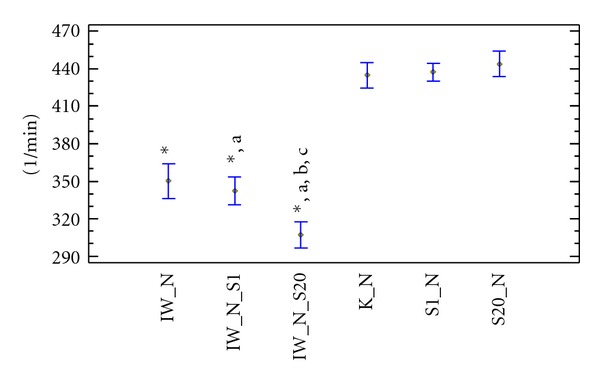
Resting mean heart rate (min^−1^) in Wistar rats fed normocholesterolaemic diet. K_N: normocholesterolaemic control group, IW_N: normocholesterolaemic group receiving ivabradine, S1_N: normocholesterolaemic group receiving simvastatin at a dose of 1 mg × kg^−1^ bw, S20_N: normocholesterolaemic group receiving simvastatin at a dose of 20 mg × kg^−1^ bw, IW_N_S1: normocholesterolaemic group receiving ivabradine and simvastatin at a dose of 1 mg × kg^−1^ bw, IW_N_S20: normocholesterolaemic group receiving ivabradine and simvastatin at a dose of 20 mg × kg^−1^ bw, *P < 0.05 as compared to the control group, (a)  *P* < 0.05 as compared to rats receiving simvastatin alone, (b)  *P* < 0.05 as compared to rats receiving ivabradine alone, and (c) *P* < 0.05 as compared to rats receiving ivabradine and simvastatin at a dose of 1 mg × kg^−1^ bw.

**Figure 4:**
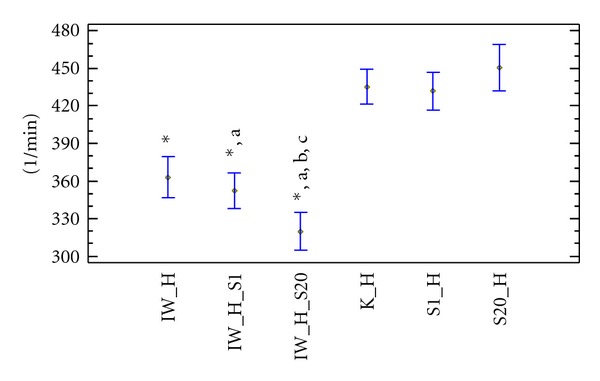
Resting mean heart rate (min^−1^) in Wistar rats fed hypercholesterolaemic diet. K_H: hypercholesterolaemic control group, IW_H: hypercholesterolaemic group receiving ivabradine, S1_H: hypercholesterolaemic group receiving simvastatin at a dose of 1 mg × kg^−1^ bw, S20_H: hypercholesterolaemic group receiving simvastatin at a dose of 20 mg × kg^−1^ bw, IW_H_S1-hypercholesterolaemic group receiving ivabradine and simvastatin at a dose of 1 mg × kg^−1^ bw, IW_H_S20: hypercholesterolaemic group receiving ivabradine and simvastatin at a dose of 20 mg × kg^−1^ bw, *P < 0.05 as compared to control group, (a) *P* < 0.05 as compared to rats receiving simvastatin alone, and (b) *P* < 0.05 as compared to rats receiving ivabradine alone, (c) *P* < 0.05 as compared to rats receiving ivabradine and simvastatin at a dose of 1 mg × kg^−1^ bw.

In hypercholesterolaemic rats, the levels of PAI-1 in the control group as well as the group receiving 1 mg ×  kg^−1^ bw simvastatin were comparable. The administration of ivabradine or 20 mg × kg^−1^ bw simvastatin to hypercholesterolaemic rats significantly lowered PAI-1 values (*P* < 0.05) compared to the control group. Concomitant administration of ivabradine with 1 or 20 mg × kg^−1^ bw simvastatin, to hypercholesterolaemic rats, did not exacerbate the fall in PAI-1 levels compared to the group receiving only ivabradine (Figure 2).

## 4. Discussion

PAI-1(serpin E1) is an inhibitor of t-Pa (tissue plasminogen activator) and u-Pa (urokinase-type plasminogen activator) and plays an important role in the regulation of activity of plasminogen. Raised levels of serum PAI-1 occur in many pathological conditions and are associated with an increased risk of cardiovascular complications [18, 19]. In an earlier study on cell cultures (HMEC and HUVEC), it was demonstrated that simvastatin significantly lowers the level of PAI-1. The observed effect was dose dependent, and the mechanism of action most probably involved the pleiotropic statin pathway [15]. The influence of simvastatin on PAI-1 values has been confirmed by clinical trials. It has been shown that administration of 40 mg × kg^−1^ bw simvastatin for 8 weeks in patients with metabolic syndrome significantly reduced the activity of PAI-1 [14]. The reduction of PAI-1 expression after “statin” administration has been evidenced in many preclinical and clinical trials [20–22]. The mechanisms surrounding the influence of HMG-CoA on the expression PAI-1 can be diverse. They include an influence on the inflammatory process, through mitogen-activated protein (MAP), nuclease factor kappa-B(NF-kB), phosphatidylinositol 3-kinase (PI3), JNK(c-jun-N-terminal kinases), and ERK (extracellular signal-regulated kinases), as well as on the small Rho proteins [23–26]. The influence of the small Rho protein family on the regulation of PAI-1 is, however, very complex and requires further studies [27]. To the best of our knowledge, this is the first study to evaluate the influence of ivabradine on PAI-1 values. Ivabradine may influence PAI-1 values by means of reducing factors associated with the inflammation process. It has been shown in E^−/−^ mice that ivabradine modulates the inflammation process by reducing the expression of interleukin–6 (IL-6) and tumor necrosis factor *alpha* (TNF-*alpha*) cytokine mRNA, which is not observed in wild-type mice [28]. The beneficial effect of reducing the inflammation process might also be a result of the slowing of the heart rate. In fact, accelerating the heart rate is associated with raised C-reactive protein (CRP) [29, 30]. The administration of ivabradine to animals receiving high dose of simvastatin has no effect on PAI-1 values compared to the groups receiving only simvastatin. However, it has been observed that ivabradine causes the reduction of PAI-1 in animals receiving small doses of simvastatin.

The mechanism of interaction on heart rate between simvastatin and ivabradine might involve a metabolic pathway. Previous pharmacokinetic studies have revealed that simvastatin might increase ivabradine plasma concentration, and, in this way, it might influence the pharmacological activity of ivabradine [31]. Although simvastatin is reported to be the substrate for P450 CYP3A4, it was seen to demonstrate inhibitory activity, as well [32]. The inhibitory activity of simvastatin is significant especially for the lactone forms rather than its acid forms [33]. Rats do not possess the CYP3A4 isoenzyme, but its activity might be adopted by others, for example, CYP 2C11, CYP3A, and CYP2D3 [34, 35]. In our study, a dose-dependent influence of simvastatin on heart rate after concomitant administration with ivabradine was observed. After concomitant administration of a small dose of simvastatin (1 mg × kg^−1^ bw) with ivabradine, the heart rates of normo- and hypercholesterolaemic rats were compared to group receiving ivabradine alone. The administration of ivabradine with simvastatin given at a higher dose (20 mg × kg^−1^ bw) caused important drug-drug interaction and significant slowing of the heart rate as compared to ivabradine alone.

The slowing of heart rate might also be a result of *beta*-blockers therapy, however the mechanistic background is different. Only several reports indicate the possible interaction between statins and *beta*-blockers. Statins reduce the isoprenoid cholesterol intermediates and as well as dolichols, geranylgeranoic acid and farnesyl-farnesoic acid and it was shown that statin influences the *beta*-adrenergic stimulation which is connected with their impact on isoprenylation of G-protein *beta*-subunits. [36]. Additionally, it was shown that simvastatin in rats restored the sympathetic/parasympathetic balance [37]. Gentlesk et al. suggested that the impact of statins on the autonomic nervous system is most probably the effect of extralipid action of simvastatin [38].

Previous studies being performed in humans [39, 40] did not reveal apparent antiadrenergic effects of statins such as a reduction of heart rate, however. Also in our previous studies simvastatin administration during two [17] and four-week (article in press) period did not influence the heart rate and blood pressure after metoprolol injection in normo- and hypercholesterolemic rats, however. In other words, any significant statin intensification of heart rate deceleration after metoprolol administration was not observed.

Another point is if the augmentation of heart rate reduction by simvastatin might be related to influence of statin on vasodilatation with enhancement the endothelium-derived nitric oxide and elevation the cGMP levels. The impact of statins on blood pressure and possible statin vasodilatory properties have been discussed widely [12]. Among suggested pathways leading to possible vasodilatory efficacy of statins, the restoration of endothelial dysfunction, increased nitric oxide synthesis with enhancement of eNOS mRNA stabilization or decreased synthesis of endothelin-1 (ET-1) are mentioned. The described effects are cholesterol-independent or “pleiotropic” ones and are the result of, at least partially, the inhibition of Rho isoprenylation [41, 42].

## 5. Conclusion

The administration of ivabradine to normocholesterolaemic and hypercholesterolaemic rats receiving simvastatin significantly exacerbated the slowing of heart rate with no effect on blood pressure. The administration of ivabradine has been shown to demonstrate different effects on PAI-1 values depending on lipid disorders. Concomitant administration of ivabradine and simvastatin in different doses, decrease PAI-1 blood levels in normo- and hypercholesterolaemic rats. 

##  Conflict of Interests

The authors have no actual or potential conflict of interests including any financial, personal, or other relationships with other people or organizations that could inappropriately influence, or be perceived to influence, this paper.

## Figures and Tables

**Table 1:** Total cholesterol (TCH), HDL-cholesterol, LDL-cholesterol, and triglycerides (TGs) (mean ± SD) in rats fed normocholesterolaemic diet (mmol/l).

	TCH	HDL	LDL	TGs
K_N (*n* = 10)	1,48 ± 0,12	0,38 ± 0,09	0,95 ± 0,29	0,32 ± 0,13
IW_N (*n* = 9)	1,11 ± 0,03	0,34 ± 0,08	0,65 ± 0,17	0,25 ± 0,08
S1_N (*n* = 10)	1,34 ± 0,21	0,57 ± 0,05*	0,56 ± 0,14*	0,46 ± 0,14
S20_N (*n* = 7)	1,39 ± 0,14	0,69 ± 0,14*	0,44 ± 0,09*	0,56 ± 0,18
IW_N_S1 (*n* = 6)	1,53 ± 0,14	0,64 ± 0,15*	0,61 ± 0,09*	0,62 ± 0,26
IW_N_S20 (*n* = 8)	1,37 ± 0,12	0,56 ± 0,18*	0,59 ± 0,10*	0,48 ± 0,15

K_N: normocholesterolaemic control group, IW_N: normocholesterolaemic group receiving ivabradine, S1_N: normocholesterolaemic group receiving simvastatin at a dose of 1 mg × kg^−1^ bw, S20_N: normocholesterolaemic group receiving simvastatin at a dose of 20 mg × kg^−1 ^bw, IW_N_S1: normocholesterolaemic group receiving ivabradine and simvastatin at a dose of 1 mg × kg^−1 ^bw, IW_N_S20: normocholesterolaemic group receiving ivabradine and simvastatin at a dose of 20 mg × kg^−1 ^bw *P < 0.05 as compared to the control group.

**Table 2:** Total cholesterol (TCH), HDL-cholesterol, LDL-cholesterol, and triglycerides (TGs) (mean ± SD) in rats fed hypercholesterolaemic diet (mmol/l).

	TCH	HDL	LDL	TGs
K_H (*n* = 10)	8,09 ± 1,53	0,36 ± 0,09	6,25 ± 0,65	3,24 ± 0,67
IW_H (*n* = 9)	7,53 ± 1,17	0,38 ± 0,11	5,98 ± 0,82	2,57 ± 0,11
S1_H (*n* = 8)	6,35 ± 1,81	0,61 ± 0,12*	4,45 ± 0,21*	2,82 ± 0,57
S20_H (*n* = 9)	2,01 ± 0,16*	0,42 ± 0,14*	1,29 ± 0,92*	0,65 ± 0,64*
IW_H_S1 (*n* = 8)	7,25 ± 0,67	0,69 ± 0,04*	4,94 ± 0,33*	3,50 ± 0,82
IW_H_S20 (*n* = 7)	1,34 ± 0,15*	0,33 ± 0,04*	0,16 ± 0,03*	0,96 ± 0,23*

K_H: hypercholesterolaemic control group, IW_H: hypercholesterolaemic group receiving ivabradine, S1_H: hypercholesterolaemic group receiving simvastatin at a dose of 1 mg × kg^−1^ bw, S20_H: hypercholesterolaemic group receiving simvastatin at a dose of 20 mg × kg^−1^ bw, IW_H_S1: hypercholesterolaemic group receiving ivabradine and simvastatin at a dose of 1 mg × kg^−1^ bw, IW_H_S20: hypercholesterolaemic group receiving ivabradine and simvastatin at a dose of 20 mg × kg^−1^ bw *P < 0.05 as compared to the control group.

**Table 3:** Summary statistics (mean ± SD) for blood pressure (mmHg) in normocholesterolaemic rats.

	Systolic blood pressure (mmHg)	Mean blood pressure (mmHg)	Diastolic blood pressure (mmHg)
K_N	105,57 ± 2,58	93,40 ± 4,55	83,96 ± 2,23
IW_N	106,66 ± 2,93	93,47 ± 3,27	85,97 ± 2,25
S1_N	104,53 ± 3,05	93,50 ± 3,13	84,63 ± 2,85
S20_N	106,79 ± 3,44	93,56 ± 5,33	86,92 ± 3,17
IW_N_S1	105,97 ± 4,37	93,51 ± 4,10	85,48 ± 3,35
IW_N_S20	106,07 ± 5,12	92,95 ± 3,52	84,53 ± 2,82

K_N: normocholesterolaemic control group, IW_N: normocholesterolaemic group receiving ivabradine, S1_N: normocholesterolaemic group receiving simvastatin at a dose of 1 mg × kg^−1^ bw, S20_N: normocholesterolaemic group receiving simvastatin at a dose of 20 mg × kg^−1^ bw, IW_N_S1: normocholesterolaemic group receiving ivabradine and simvastatin at a dose of 1 mg × kg^−1^ bw, IW_N_S20: normocholesterolaemic group receiving ivabradine and simvastatin at a dose of 20 mg × kg^−1^ bw.

**Table 4:** Summary statistics (mean ± SD) for blood pressure (mmHg) in hypercholesterolaemic rats.

	Systolic blood pressure (mmHg)	Mean blood pressure (mmHg)	Diastolic blood pressure (mmHg)
K_H	107,77 ± 3,80	94,96 ± 3,73	85,86 ± 2,64
IW_H	105,46± 2,00	92,63 ± 2,75	86,38 ± 2,19
S1_H	105,47 ± 2,82	93,91 ± 4,14	85,20 ± 3,15
S20_H	106,81 ± 4,01	94,04 ± 3,20	85,32 ± 3,79
IW_H_S1	105,47 ± 3,40	94,48 ± 4,19	86,48 ± 3,42
IW_H_S20	105,57 ± 3,43	93,95 ± 2,28	86,24 ± 4,33

K_H: hypercholesterolaemic control group, IW_H: hypercholesterolaemic group receiving ivabradine, S1_H: hypercholesterolaemic group receiving simvastatin at a dose of 1 mg × kg^−1^ bw, S20_H: hypercholesterolaemic group receiving simvastatin at a dose of 20 mg × kg^−1^ bw, IW_H_S1: hypercholesterolaemic group receiving ivabradine and simvastatin at a dose of 1 mg × kg^−1^ bw, IW_H_S20: hypercholesterolaemic group receiving ivabradine and simvastatin at a dose of 20 mg × kg^−1^ bw.

## Abbreviations

AUC: Area under plasma concentration time curve
Bw: Body weight
CAD: Coronary artery disease
CRP: C-reactive Protein
ET-1: Endothelin-1
ERK: Extracellular signal-regulated kinases
HDL: High density lipoprotein cholesterol
HMGCo-A reductase: 3-hydroxy-3-methyl-glutaryl-CoA reductase
Ip: Intraperitoneal
IHD: Ischemic heart disease
JNK: C-jun-N-terminal kinases
LDL: low density lipoprotein cholesterol
LVSD: Left ventricular systolic dysfunction
MAP: Myogen-activated protein
NO: Nitric oxide
NF-kB: Nuclease factor kappa-B
PI3: Phosphatidylinositol 3-kinase
TCH: Total cholesterol
TG: Triglycerides
TNF-*alpha*: Tumor necrosis.
